# Supplementary material for: Polyamines in Edible and Medicinal Fungi from Serbia: A Novel Perspective on Neuroprotective Properties
Source: J Fungi (Basel). 2023 Dec 28;10(1):21. doi: 10.3390/jof10010021 (PMC10816940; doi:10.3390/jof10010021)
Supplement: Supplementary file 1 [file jof-10-00021-s001.zip › jof-2764299-supplementary.pdf]

Table S1. Results of CHN analysis.

	N (%)	C (%)	H (%)
Acetanilide	10.322±1.52	70.728±4.25	6.671±0.85
Clitocybe odora	6.352±0.85	39.833±3.33	7.058±1.05
Clitopilus prunulus	7.472±0.66	38.961±2.95	6.850±0.94
Lepista nuda	7.585±0.94	39.317±2.38	6.930±1.12
Postia caesia	2.979±0.13	40.830±3.01	6.751±1.35
Morchella elata	5.539±0.51	37.860±2.77	6.647±0.98
Cyclocybe aegerita	4.843±0.37	39.979±2.89	6.996±0.85
Ganoderma applanatum	2.759±0.35	42.669±2.91	6.035±1.45
Ganoderma resinaceum	2.182±0.35	40.373±1.56	6.210±0.51
